# Hanna’s Modified Sagittal Split Osteotomy (HSSO): An Alternative to Inverted L Osteotomy—Merging Function and Aesthetics for Enhanced Stability, Attractiveness, and Nerve Protection

**DOI:** 10.3390/jcm13123438

**Published:** 2024-06-12

**Authors:** Todd Hanna, Ketan Bansal, Robert Radu Ilesan, Daniel Buchbinder

**Affiliations:** 1 Private Practice, Hanna Face and Jaw PC, New York, NY 10029, USA; dr.hanna@hannafacejaw.com; 2 Division of Oral and Maxillofacial Surgery, Department of Otolaryngology-Head and Neck Surgery, Icahn School of Medicine at Mount Sinai, Mount Sinai Beth Israel, New York, NY 10029, USA; ketanbansal26@gmail.com (K.B.);; 3 Department of Oral and Maxillofacial Surgery, Albert Einstein College of Medicine, Jacobi Medical Center, Bronx, NY 10461, USA; 4 Department of Maxillofacial Surgery, ZMACK, AZ MONICA Antwerp, Harmoniestraat 48, 2018 Antwerp, Belgium; 5 Department of Cranio-Maxillofacial Surgery, Antwerp University Hospital, Wilrijkstraat 10, 2650 Edegem, Belgium; 6 Faculty of Medicine & Health Sciences, University of Antwerp, Campus Drie Eiken, Universiteitsplein 1, 2610 Antwerp, Belgium

**Keywords:** Hanna’s modified sagittal split osteotomy, inverted L osteotomy, transoral inverted L, dentofacial deformities, mandibular asymmetry, virtual surgical planning, hemifacial hypertrophy, hemifacial microsomia, idiopathic condylar resorption, aesthetic orthognathic surgery

## Abstract

**Background**: The current high standards in orthognathic surgery demand surgical solutions that are both ⁠ functionally ⁠ effective and aesthetically pleasing. Our approach offers one for enhanced stability, attractiveness, and nerve protection ⁠ with improved accessibility ⁠ in the majority of orthognathic scenarios ⁠ compared to an inverted L osteotomy. **Methods:** A case series is presented to illustrate the application and outcomes of HSSO, an optimised approach that combines the advantages of a transoral inverted L osteotomy with specific enhancements and increased versatility, ⁠ with accessibility and exposure similar to a BSSO. **Results:** HSSO as a completely transoral technique, demonstrate the ability to perform significant counterclockwise rotations of the mandible, eliminating the need for trocars or skin incisions. We experinced high postoperative stability when HSSO was performed in conjunction with a three-piece LeFort 1 osteotomy on a dynamic opposing arch. In comparison to an inverted L approach, we postulated that HSSO offers advantages in stability, due to the increased segmental overlap of the proximal and distal segments of the mandible. This approach is designed to enhance the safety of the inferior alveolar nerve compared to traditional sagittal split methods. Furthermore, HSSO represents an alternative to total joint replacement in select cases of idiopathic condylar resorption and is effective for correcting mandibular asymmetries while maintaining jawline aesthetics. This is achieved through the manipulation of the mandibular angle, ramus height, and inferior border without creating a step deformity in the soft tissue. **Conclusions:** The outcomes of HSSO highlight its capacity to deliver predictable, functional, and aesthetically pleasing results, offering a viable alternative to more traditional orthognathic techniques.

## 1. Introduction: A Historical Perspective and Milestones in Orthognathic Surgery—Bilateral Mandibular Split Osteotomy

Orthognathic surgery has its roots in the mid-19th century with the introduction of procedures specific for either mandibular advancement, setback, or asymmetry and deformities [1]. It all began in 1849 when Simon P Hullihen published a paper detailing the first mandibular osteotomy to correct a skeletal anterior open bite resulting from scar contractures due to a facial burn [2]. This was technically a bilateral bicuspid region wedge ostectomy to “set back” the anterior mandibular dentoalveolar segment.

**Figure 1:**
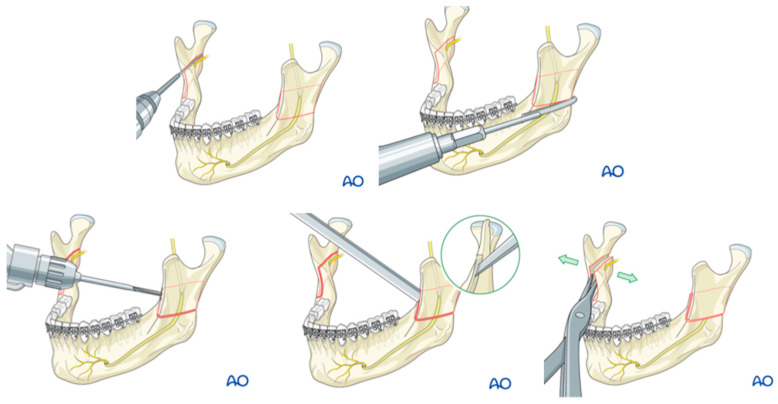
Illustrations demonstrating the first sagittal splitting of the rami [15].

Nearly half a century after initial attempts to address mandibular prognathism, James Whipple documented the long-term observation of ‘Mr. K’ in 1898, noting his pronounced lower jaw protrusion. Consulting with Edward H. Angle, pioneer of modern orthodontics, they proposed a revised Hullihen’s procedure to correct this [1,3]. However, in the following year of 1897, Vilray Papin Blair performed the proposed surgery with a slight modification. Blair opted for parallel osteotomies rather than the angled ones initially proposed by Angle himself. Four months later, Whipple fitted gold crowns on the patient’s posterior teeth to enhance occlusion. Whipple published the case report titled “Double resection of inferior maxilla for protruding lower jaw” in Blair’s absence [4,5,6,7]. In 1917, Blair [8] introduced a method for treating prognathism, which involved a blind transcutaneous osteotomy of the ramus above the lingula, performed with a gigli saw. This approach was further refined into a horizontal osteotomy by Kostecka [9] in 1931 and became one of the primary methods for addressing prognathism at that time. Until the mid-20th century, orthognathic surgery was constrained in its applications, with each procedure tailored specifically to address either mandibular advancement, setback, asymmetry, or deformities [1]. This was until the introduction of the intraoral sagittal split osteotomy in 1955 by Trauner and Obwegeser [10,11,12,13], Figure 1, which allows manipulation of the mandible in all three planes. They developed this technique following complications in more than 50% of the 36 cases of prognathism they treated using Kostecka’s procedure [14]. Trauner theorized that the occurrence of partial and complete relapse could be attributed to insufficient bony contact between the two mandibular segments. Consequently, the pair sought to develop a completely transoral technique that increased the bony contact area and protected the contents of the mandibular canal [14].

**Figure 2:**
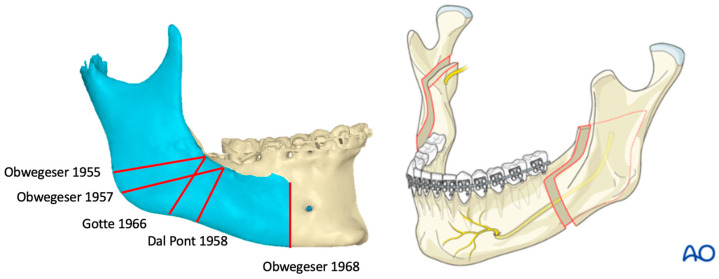
Illustrations demonstrating the Dal Pont modification for the sagittal splitting of the rami [15].

In 1957, while observing under the guidance of Dr. Obwegeser, Dal Pont introduced a notable modification to the osteotomy technique, transitioning the lateral osteotomy from a horizontal to a vertical orientation [16,17]. In doing so, Dal Pont significantly increased the bone-to-bone contact, resulting in a substantially greater connection between the two segments, Figure 2. This also facilitated more effective advancement of the mandible’s distal segment, as detailed in his 1958 publication [16].

**Figure 3:**
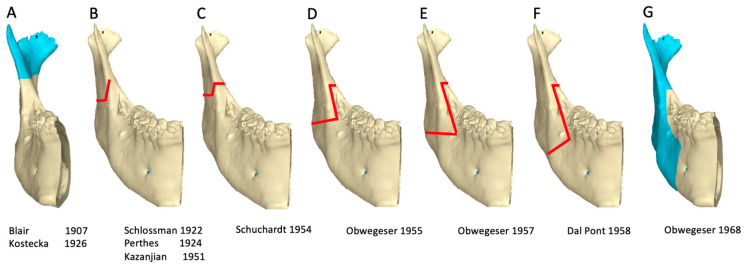
Evolution of increasing bone-to-bone contact in mandibular osteotomies [10]. (**A**): Blair (1907). (**B**): Schlossmann–Perthes–Kazanjian (1922–1951). (**C**): Schuchardt (1954). (**D**): Obwegeser (1955). (**E**): Obwegeser (1957). (**F**): Dal Pont (1958). (**G**): Obwegeser (1968). The dates indicate the publication and not the date of the first procedure by the surgeon [10].

It should be noted that Schuchardt’s 1954 publication was based on his experience when he assisted Obwegeser with a transoral sagittal splitting of the ramus on April 22, 1953 [10]. Furthermore, Dal Pont’s publication in 1958 showed photos of a patient of Obwegeser and that the procedure was performed when Dal Pont was a trainee at Zurich under Obwegeser, Figure 3 [10].

In 1961, the next modification to this technique was recommended by Dal Pont [18] as well as Hunsuck and Epker in 1968 [19,20,21,22,23]. They noted that an incomplete split of the medial portion that did not extend to the posterior border of the ramus still produced satisfactory results at which point they “advocated that the osteotomy of the lingual side should be incomplete, extending just past the entrance of the neurovascular bundle” [10]. A truly remarkable milestone during this period was the introduction of plate and screw fixation to achieve compression at the osteotomy site. This technique, which was pioneered by Hans-Georg Luhr in 1968, has since revolutionized surgical outcomes [1,24,25]. In the late 1980s, Posnick used a modified medial osteotomy positioned just above the mandibular molar occlusal plane that extends below the lingula [23,26]. This modification progresses posteriorly for a maximum of 2 cm and has the advantage of effectively reducing posterior interference between the proximal and distal segments, which is often seen in cases of asymmetry [27,28,29]. In 1987, Wolford introduced a modification to increase bone-to-bone contact through the implementation of a stepwise osteotomy on the buccal cortex [30]. Wolford and Davis further refined the technique in 1990 with the introduction of an inferior border osteotomy and specifically designed saw blades for the osteotomy [31,32,33].

The evolution of these techniques over time has culminated in their widespread adoption to address mandibular conditions including hypoplasia, hyperplasia, and asymmetry. Presently, practitioners frequently utilize modified iterations of these methods, collectively referred to as the bilateral sagittal split osteotomy (BSSO) in contemporary practice. Maxillofacial surgery was among the first specialties to incorporate 3D planning and printing into its standard practices, significantly improving surgical precision and patient outcomes [34,35,36]. Furthermore, promising results have indicated that the near future of orthognathic surgery workflows may include the integration of virtual reality [37] and robotic laser osteotomies [38], reshaping personalized treatment. In our orthognathic surgery workflows, we have already standardized the use of VSP and patient-specific implants (PSIs). We are optimistic that ongoing research will facilitate the incorporation of augmented reality, further enhancing our surgical procedures. We sought to spotlight the major advancements in mandibular split osteotomy and introduce a refined variant of the technique, ushering in the era of customized osteotomies. We welcome feedback from our readers.

### Publication Aim

This surgical technique adaptation aims to rectify several issues inherent in the conventional method. This improvement synergizes fundamental components of aesthetic and maxillofacial surgery, achieving a cohesive fusion of functionality and aesthetic values. The anticipated benefits of this technique include enhanced stability, improved aesthetic results, and better nerve preservation, as highlighted in the next case narratives.

## 2. Surgical Technique

Before orthognathic surgery, a thorough pre-operative protocol is vital for success, echoing Benjamin Franklin’s adage, “if you fail to plan, you are planning to fail”. The process starts with a patient interview and psychological evaluation to gauge their concerns, expectations, and surgical readiness. We then gather comprehensive information on their medical background to assess overall health. A thorough maxillofacial examination is conducted to evaluate facial structure, occlusion, jaw movements, dental health, aesthetics, and temporomandibular joint functionality. We address any orthodontic needs, assess speech and swallowing functions, and screen for obstructive sleep apnea, referring to a sleep study if needed. We also collect detailed visual records using intraoral and extraoral photography, video analysis, and digital impressions. Advanced imaging techniques like CT/CBCT scans and virtual surgical planning enhance surgical accuracy. Patients receive both verbal and written informed consent with 3D demonstrations of expected outcomes. In the lead-up to the operation, patients are guided on essential protocols including orthodontic care, medications, dietary adjustments, necessary lab tests, and lifestyle changes, with a particular focus on smoking cessation as required. 

**Figure 4:**
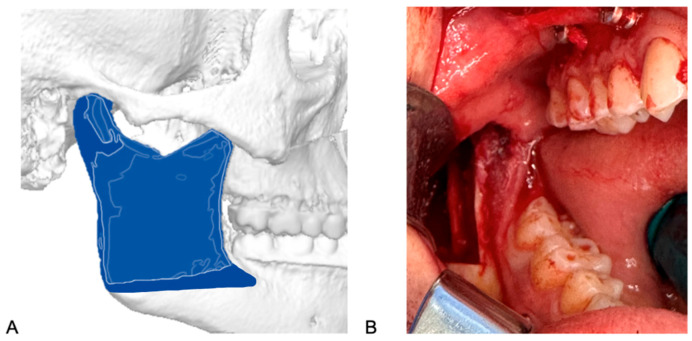
(**A**): Cutting line of Hanna’s osteotomy. (**B**): Intraoral view of osteotomy and protection of soft tissue using Langenbeck toe-out retractor.

In Hanna’s modified sagittal split osteotomy (HSSO, Figure 4B), the patient is placed in a supine position and administered general anesthesia via nasotracheal intubation. The endotracheal tube is secured to the head dressing, with care to avoid nasal distortion. For this reason, a nasal RAE (Ring–Adair–Elwyn) tube is used. A sterile prep and drape is performed, along with review of virtual surgical planning and materials. The HSSO is typically carried out in conjunction with a LeFort osteotomy and genioplasty, which will not be discussed. A local anesthetic with vasoconstrictor, such as 2% lidocaine with 1:100,000 epinephrine, is administered via inferior alveolar, lingual, and long buccal nerve blocks as well as submucosal infiltration anteriorly into the buccal vestibule and posteriorly along the ascending ramus. A bite block is placed on the contralateral side followed by a tongue retractor on the working side. A retractor is placed firmly into the buccal vestibule lateral to the external oblique ridge at the anterior border of the ramus to accentuate the external oblique ridge. Utilizing either a 15 blade or electrocautery, a standard mucosal incision is initiated approximately halfway up the anterior border of the ramus at the external oblique ridge. Following the external oblique ridge, the incision is carried inferiorly to the second molar where it continues just lateral to the external oblique ridge up to the distal of the first molar to maintain a cuff of tissue by the teeth (medial to the incision) to facilitate closure. The incision is completed to full thickness through the submucosa, muscle, and periosteum down to bone. With a periosteal elevator, the tissue is then dissected in a subperiosteal plane anteriorly to the mesial of the first molar, posteriorly to the gonial angle, and inferiorly stopping prior to the inferior border, maintaining the inferior border attachments (pterygomasseteric sling) and good vascularity, thereby revealing the lateral aspect of the ramus. A small area of elevation is implemented at the inferior border to accommodate the surgical guide when custom hardware is being used. At this stage, in selected cases, such as hyperdivergent profiles, we may elect to cut the sling along with the insertion of the sphenomandibular ligament to help minimize relapse [39]. A V-notch retractor or ramus stripper is then placed along the external oblique ridge and, using controlled apical pressure, is pulled superiorly up to the coronoid process exposing the attachments of the temporalis muscle. A periosteal elevator is used to partially release these attachments allowing for visualization and access to the medial ramus. Using a blunt elevator, subperiosteal dissection is continued along the medial aspect of the ramus, beginning superiorly and extending inferiorly and posteriorly until the lingula is visible. The lingula is typically located approximately 1 cm above the occlusal plane and 3 cm posterior to the second molar [40]. The lingual nerve is reflected and protected with the proximal tissues using a blunt elevator. Once the nerve is adequately protected, a Seldon retractor is used to provide lateral retraction. A reciprocating saw or piezoelectric handpiece is then placed on the medial aspect of the ramus parallel with the occlusal plane, superior and posterior to the lingula, and the horizontal osteotomy is carried out through cortical bone to the depth of cancellous bone. Employing a reciprocating saw, or piezoelectric handpiece, a sagittal osteotomy then progresses anteriorly along the ascending ramus to the depth of cancellous bone, approaching the external oblique ridge and halting distal to the first molar. At this point, the bite block is removed from the contralateral side and, if utilized, the custom cutting guide is placed and secured with two or three fixation screws. The predictive holes are drilled with a straight or right-angle driver under saline irrigation. With the guide in place, the remainder of the sagittal osteotomy is made, followed by the outline of the oblique or low-horizontal osteotomy. The guide is then removed, and the osteotomy is carried to the posterior ramus above the gonial angle using a Langenbeck toe-out retractor to protect the soft tissue, Figure 4B.

**Figure 5:**
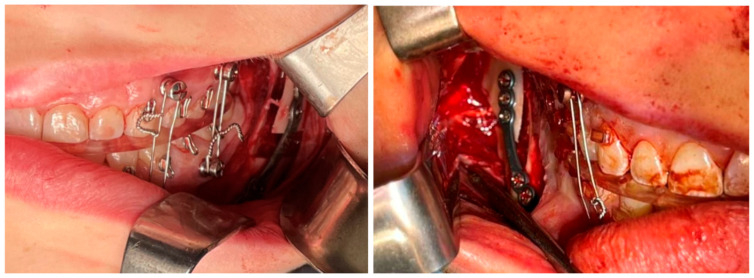
Intraoperative image of Hanna’s osteotomy, MMF, and osteosynthesis with patient-specific implants.

The subsequent split adheres to traditional techniques, employing straight and curved chisels, osteotomes, wood-handled osteotomes, and/or Tessier and Smith spreaders. If necessary, a wedge is removed from the proximal segment rami using a reciprocating saw to shorten the ramus. This would be designed into the cutting guide as depicted in Case 3. The procedure is mirrored on the contralateral side, and the patient is then placed in maxillomandibular fixation (MMF) with an occlusal splint, Figure 5. Ensuring the condyles are seated appropriately, the mandibular segments are fixated, and if a patient-specific plate is utilized, a right-angle driver is recommended for ease of accessibility.

The bite block and tongue retractor are removed, and occlusion is confirmed prior to copious irrigation and standard closure. The oropharynx is cleared through suction. A nasogastric tube may be passed to further clear any excess fluid and the care of the patient is handed back to the anesthesia team. 

Post-operative care will be standard to traditional BSSO in guided cases. In non-guided cases, orthodontic treatment must be maintained for a slightly longer period, and we must refrain from using anterior maxilla to posterior mandible elastic vectors for 4–6 months due to the leverage created at the angle. Follow up occurs at 1 week, 2 weeks, 4 weeks, 3 months, 6 months, and 12 months post-operatively.

## 3. Practitioners Result: A Closer Look at Three Cases

### 3.1. Case 1

**Figure 6:**
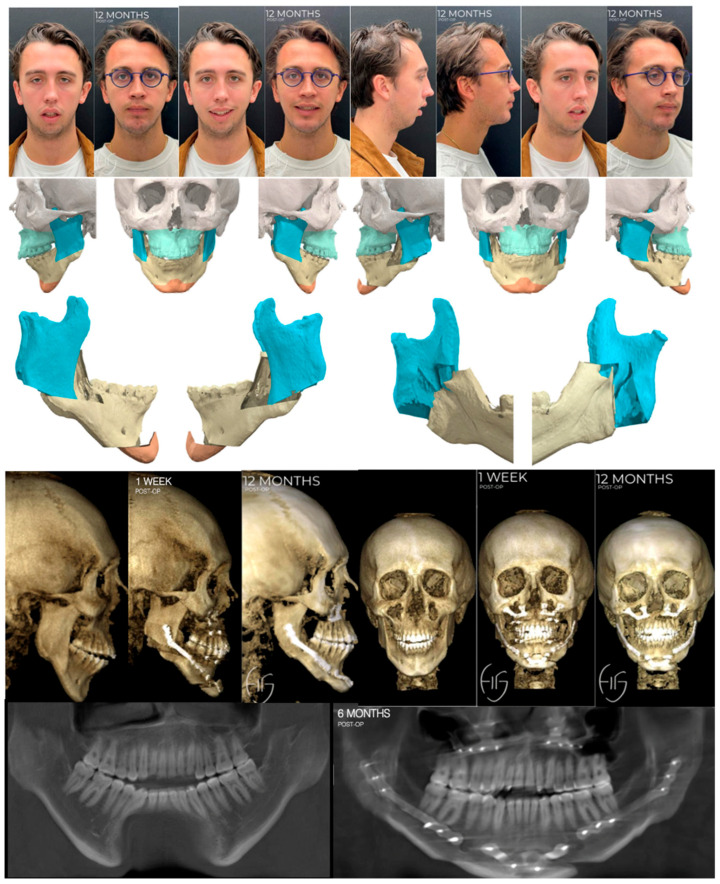
Custom approach utilizing HSSO for large counterclockwise rotation to correct a hyperdivergent profile with concomitant bilateral idiopathic condylar resorption. At the time of writing this article, the patient is currently stable 13 months post-Le Fort 1, HSSO, and genioplasty without any complications, changes in occlusion, or relapse.

The first case depicts a healthy 21-year-old male patient who sought care for a severe anterior open bite. Following our standard pre-surgical workflow, the patient was diagnosed with a maximal incisal opening of approximately 35–40 mm without pain in the temporomandibular joints, a hyperdivergent profile (downward rotation of the maxillomandibular complex relative to the anterior cranial base), and concurrent non-active bilateral total idiopathic condylar resorption. Treatment options included bilateral total joint replacement or an orthognathic approach using the HSSO in conjunction with a single piece Le Fort 1 and genioplasty. An orthognathic approach is a viable option as an alternative to total joint replacement in idiopathic condylar resorption cases where there is adequate maximal incisal opening and no pain in the temporomandibular joints [41]. We chose to pursue an orthognathic approach, acknowledging the possibility of relapse due to continued idiopathic condylar resorption, as shown in Figure 6. However, given the minimal remaining condyles left for potential resorption, any progression was expected to be more conservative, with less clinical impact. Additionally, in case of relapse, the option of total joint replacement remained available.

This case holds particular interest, notably due to the decreased susceptibility of males in comparison to adolescent females, particularly among cheerleaders, to idiopathic condylar resorption with a 1:9 frequency ratio [42]. This patient presented with a hyperdivergent profile which usually presents with a concomitant long lower facial third, short ramus height, steep mandibular plane angle, and anterior open bite [43]. To correct this, a large counter-clockwise rotation of the maxillo-mandibular complex was required. This can be achieved with a Le Fort 1 and mandibular osteotomy, of which the options of BSSO, Inverted-L [11], intraoral vertical ramus osteotomy (IVRO) [44], and now HSSO exist. The BSSO is versatile, but it has its limitations. These include large setbacks greater than 7mm [45] or advancements greater than 10 mm [46,47,48,49], large vertical changes, and large rotational changes [50,51,52,53] such as that required in this case. These limitations can be addressed by use of the inverted L or IVRO osteotomies, which have the primary drawback of a Risdon [54] (transfacial submandibular skin) incision. Franco and Farrell [55] describe a solution to this with the introduction of the intraoral inverted L procedure in 2016 that utilized virtual surgical planning (VSP), thereby eliminating the need for a Risdon incision but still utilizing a trocar. The HSSO incorporates all the advantages of the intraoral inverted L procedure without the requirement for a trocar or VSP and shows theoretic improved stability due to increased segmental overlap of the proximal and distal segments of the mandible in comparison to an inverted L. It also eliminates the risk of damage to the facial artery and vein by avoiding an osteotomy through the inferior border at the antegonial notch. For these reasons, we elected to proceed with the HSSO over the other mandibular osteotomies in this case.

An advantage to the HSSO is that it can be performed with, Cases 1 and 3, or without, Case 2, a surgical guide. For Case 1, we decided to use a virtual surgical planning workflow with surgical guides and PSIs by KLS Martin. This was because the absence of condyles makes repositioning of the proximal segments less predictable as manual repositioning remains the method of choice [56] and relies on both the tactile seating of the condyle and the experience of the surgeon. As a result, there exists an empiric tendency towards over-rotation of the proximal segment. Surgical guides and PSIs allow us to overcome this and control the posterior and lateral positioning of the proximal segment, ensuring equal width bilaterally and adequate seating of the condyles. Additional advantages of guided surgeries are that they have shorter operating times [34], decreased blood loss [57], and increased stability due to the use of milled titanium plates and longer fixation screws relative to mini-plate fixation [58]. Moreover, they provide increased patient education and informed consent, as the surgery can be visualized in 3D [59,60,61].

The limitations of guided surgery in HSSO may involve the need for more extensive dissection due to surgical guides. It is important to note that guided surgery typically incurs higher costs [62] and is best performed by experienced surgeons to manage potential intraoperative complications that could require a shift to traditional methods. Additionally, the adoption of guided surgery in HSSO may present an increased learning curve. In specific instances of guided HSSO, the use of a right-angle driver is essential for plate fixation.

### 3.2. Case 2

**Figure 7:**
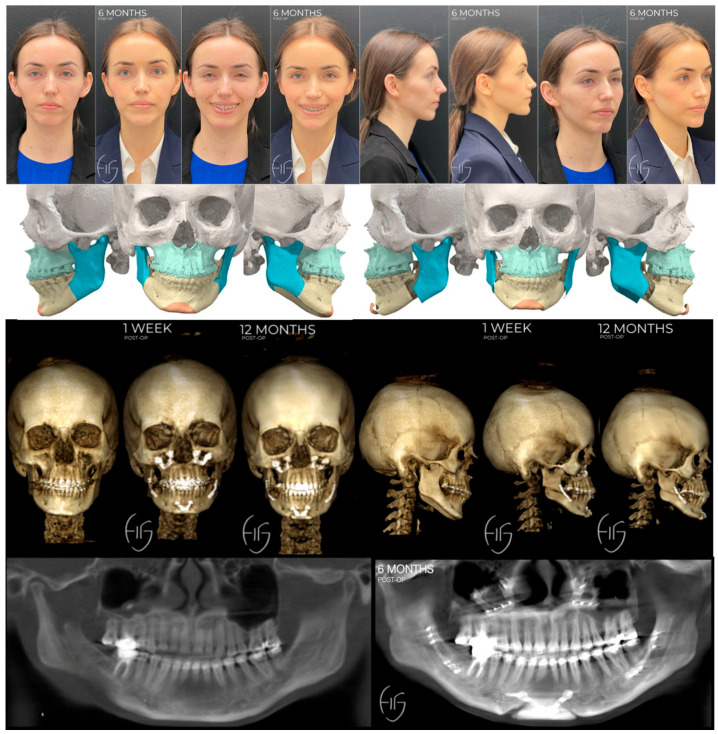
Non-custom approach utilizing HSSO to elongate the ramus and correct mandibular asymmetry. VSP to demonstrate residual step deformity and asymmetry at angle and inferior border following traditional BSSO. At the time of writing this article, the patient is currently stable 12 months post-Le Fort 1, HSSO, genioplasty, rhinoplasty, and bilateral otoplasty without any complications, changes in occlusion, or relapse.

**Figure 8:**
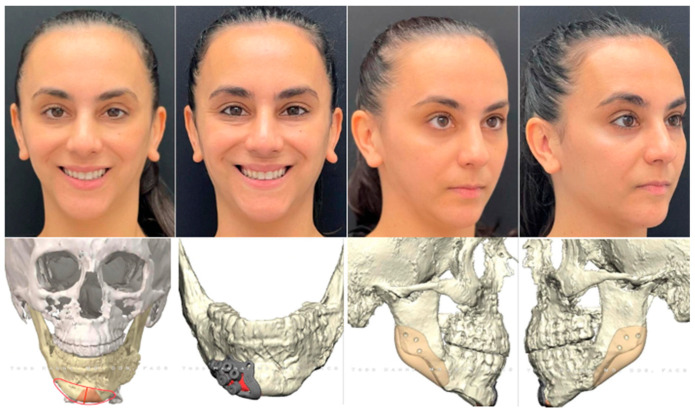
Residual asymmetry and contour deformity post-orthognathic surgery. Images are pre-operative and 3 months post-correction via bilateral PEEK angle onlay implants and revised genioplasty.

**Figure 9:**
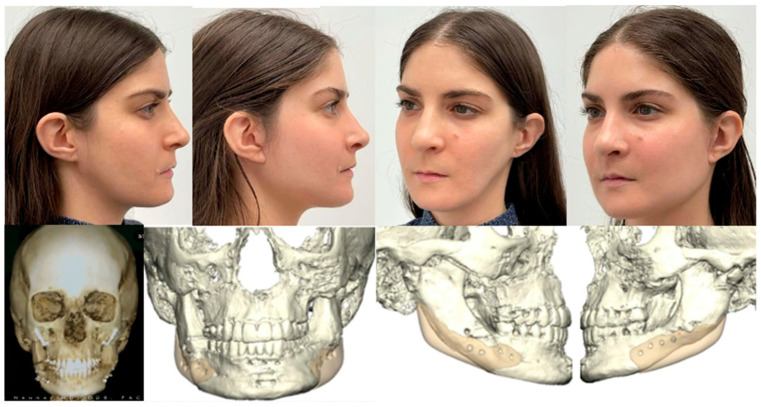
Residual asymmetry and contour deformity post-orthognathic surgery. Images are pre-operative and 3 months post-correction via bilateral PEEK angle onlay implants, subnasal lip lift, and mini face lift.

The second case depicts a healthy 30-year-old female patient who was referred for management of a skeletal and dental class 2 deformity with asymmetry and had aesthetic concerns of the nasal dorsum and bilateral ears which are enlarged at the conchal bowl. Following our standard pre-surgical workflow, the patient was diagnosed with class 2 skeletal and dental deformity, rightward facial asymmetry, maxillary cant, and malocclusion producing severe myofascial pain. She also presented with under contoured inferior borders, nasal dorsal deviation, and bilateral otapostasis (prominent ears or bat ear deformity). Treatment options for the mandibular asymmetry included a traditional BSSO +/− PEEK (polyether ether ketone) angle onlay PSIs or a HSSO in conjunction with the Le Fort 1, genioplasty, rhinoplasty, and otoplasty needed for her other diagnoses. We chose the HSSO as it allows us to correct the mandibular asymmetry by elongating the ramus while maintaining jawline aesthetics, as shown in Figure 7. This is achieved via manipulation of the mandibular angle and avoidance of a step deformity in the inferior border, mitigating the need for angle onlay implants. Inferior border step deformities can project into the soft tissue, a complication associated with traditional BSSO procedures as depicted in the demonstrative VSP in Figure 7 and the two cases shown in Figure 8 and Figure 9. Avoiding this complication is particularly beneficial in this case as the patient naturally exhibits an under contoured inferior border, which would be accentuated by soft tissue projection of a step deformity at the inferior border. In HSSO, the step deformity will be at the posterior border of the ramus above the gonial angle. This site is masked by the masseter and parotid gland, mitigating the risk of soft tissue projection that could occur with inferior border positioning of the bony step.

Figure 8 and Figure 9 depict the soft tissue projections of the step at the inferior border following a traditional BSSO. These residual asymmetries and contour deformities are particularly prevalent in cases resulting in a vertical discrepancy between the proximal and distal segments of the mandible. Correction requires the use of patient-specific angle onlay implants [63,64,65]. PEEK implants are typically used for this due to the low complication rate reported in the literature [66]; however, when complications do arise and the prosthesis must be removed, it can be very challenging due to the porous property of the material [67]. Consequently, we posit that HSSO represents an optimal alternative to traditional BSSO and a viable option in repositioning bony landmarks for angle and jawline contouring.

Further advantages of the HSSO, beyond those previously mentioned, include the maintenance of the intergonial distance, as the gonial angles are part of the distal segment and remain connected within the mandibular arch. This contrasts with a traditional BSSO and PEEK angle PSIs. In a traditional BSSO, the gonial width may change as the angles are part of the proximal segment which can swing laterally at the TMJ causing widening of the lower facial third [68,69,70]. The addition of a PEEK angle PSI will inherently cause widening of the gonial width as it adds a foreign body. Therefore, HSSO is particularly advantageous for female patients who typically prefer a narrower lower facial third. As mentioned in Case 1, an advantage of HSSO is that it can be completed with or without a surgical guide and PSIs. This case was virtually planned but the osteotomy was completed without a guide and fixated using stock alloy miniplates, utilizing a straight screwdriver. By doing this, we maintained the inferior border periosteum with a minimally invasive approach that minimizes dissection and does not require a right-angled driver. Lastly, by opting for HSSO instead of BSSO and PEEK angle PSIs, we minimize the complications associated with the latter two.

A notable drawback of non-guided HSSO surgery is the potential for decreased stability, which may result in leverage being exerted at the osteotomy site near the angle. In a worst-case scenario, these factors combined could precipitate the development of an anterior open bite, which would require either revision surgery or orthodontic management.

### 3.3. Case 3

The third case depicts a healthy 18-year-young female patient who sought care for congenital right-sided facial asymmetry. Following our standard pre-surgical workflow, the patient was diagnosed with right-sided hemifacial hypertrophy resulting in maxillo-mandibular asymmetry with an elongated right ramus height and over-contoured right inferior border. She also exhibited slight asymmetries at the zygoma and malar bones, along with fatty and soft tissue imbalances.

**Figure 10:**
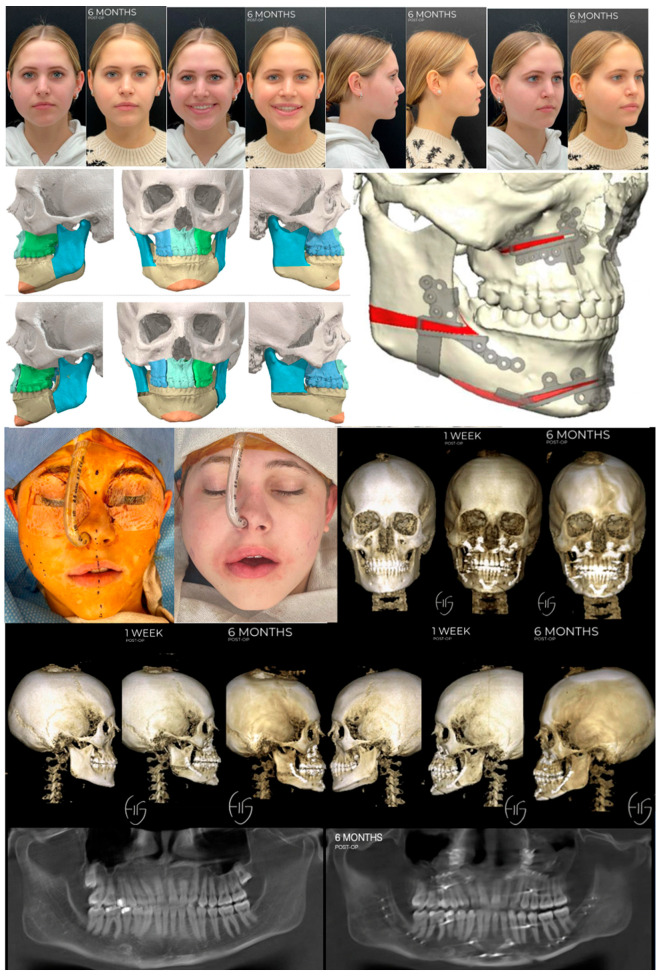
Custom approach utilizing HSSO and traditional BSSO to shorten the ramus and raise the inferior border on one side to correct hemifacial hypertrophy. At the time of writing this article, the patient is currently stable 12 months post-three-piece Le Fort 1, right-sided HSSO, left-sided BSSO, and genioplasty without any complications, changes in occlusion, or relapse. The patient is planned for a secondary surgery to manage the right-sided soft-tissue excess.

**Figure 11:**
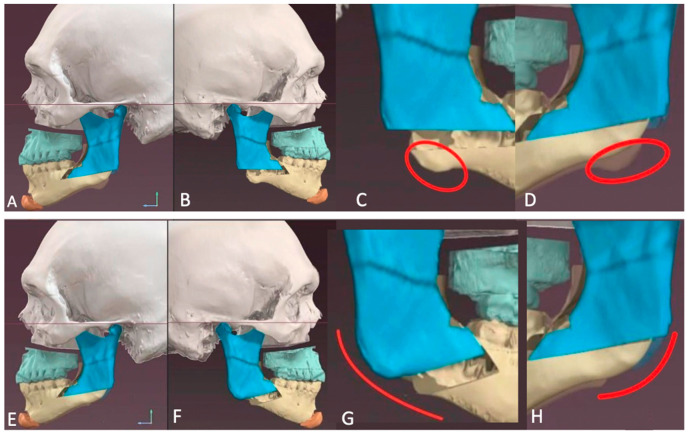
Virtual workflow demonstrating difference in gonial angle, antegonial notch, and inferior border projection with (**A**–**D**): low horizontal osteotomy superior to angle vs. (**E**–**H**): oblique osteotomy anterior to angle.

The patient’s surgical plan involved a three-piece Le Fort, genioplasty, right buccal fat pad reduction, and correction of mandibular asymmetry, followed by a secondary surgery to address the soft tissue imbalances. Management of the mandibular asymmetry involved reducing the right ramus height, raising the right inferior border and gonial angle while maintaining the left-side proportions. To achieve this, we chose to perform a traditional BSSO on the left side with an HSSO on the right. When the ramus height and gonial projection is adequate on one side, an osteotomy that retains the ramus and gonial angle in the proximal segment is advantageous. This can be achieved via a traditional BSSO as depicted on the patient’s left side in this case, Figure 10, or an oblique HSSO that extends anterior to the angle into the antegonial notch as depicted in Figure 11F,G. A traditional BSSO is employed when we want to maintain the position of the gonial angle and adjacent inferior border by keeping them in the proximal segment, acknowledging the risk of developing a postoperative soft tissue contour deformity. An oblique HSSO anterior to the angle is completed when we want to change the position of the inferior border and antegonial notch but maintain the projection of the gonial angle. By directing the osteotomy anteriorly, the angle and adjacent inferior border remain in the proximal segment, so their position will not change as we reposition the distal segment. Figure 11 depicts a workflow demonstrating the difference between a low-horizontal HSSO compared to an oblique HSSO. 

The advantage of performing an HSSO on the right side vs. a traditional BSSO is that the HSSO allows for complete manipulation of the ramus height and gonial projection. The HSSO provides versatility as it allows us to shorten the ramus height by removing a wedge, as performed on the right side of this case, maintain it as on the left side of this case, or elongate it as described earlier in Case 1. Due to this ability to control ramus height, HSSO may also be implemented in cases of mandibular insufficiency, such as in this case of hemifacial microsomia [71], once again potentially eliminating the need for patient-specific angle onlay implants following orthognathic surgery [71]. The wedge removal from the ramus also eliminates the need for an inferior border reduction, thereby maintaining the integrity of the inferior border. Of note, a similar wedge removal was described in a case of hypertrophy secondary to acromegaly [72] using a retromandibular approach. However, in comparison, the HSSO has the added benefit of avoiding a skin incision as mentioned previously. This case also utilized HSSO in conjunction with a three-piece Le Fort, demonstrating its stability with a dynamic opposing arch form, demonstrating both occlusal and bony stability. Lastly, this case was virtually planned, thereby incorporating the previously discussed advantages and disadvantages.

Figure 11A–D and Figure 11E–H depict a simulation of the same patient with the same low-horizontal HSSO osteotomy carried out on the left side and different osteotomies on the right side for comparison. In the first simulation, Figure 11A–D, a low-horizontal HSSO was carried out on the right side, Figure 11B. In the second simulation, Figure 11E–H, an oblique HSSO was carried out on the right side, Figure 11F. In this simulated patient, the right side has a deeper antegonial notch and a longer posterior ramus than the left side. This creates vertical asymmetry at the gonial angles as depicted by the red circles in Figure 11C,D. Directing the osteotomy anterior to the angle at the notch on the right side and keeping it above the angle on the left side aids in leveling the inferior border and achieving greater symmetry in the angles as depicted by the red lines in Figure 11G,H. This is because the entire ramus and gonial angle on the elongated right side remain in the proximal segment without alteration, while the shorter left side is now in the distal segment, allowing for adjustment of the angle to match the other side. Furthermore, positioning the oblique HSSO into the antegonial notch allows us to level the inferior border at this location. Notice the difference of antegonial notch depth and inferior border continuity on the right side between Figure 11C,G. Notice the difference in symmetry of the gonial angles between Figure 11D,H.

## 4. Always Forward Thinking

We are committed to exploring innovative surgical techniques and welcoming advancements that promise improved outcomes for our patients. This dedication propels us to critically evaluate and refine our approaches, ensuring we address current limitations while also anticipating future challenges.

The primary concern with the HSSO is the leverage created at the angle. To augment stability, the employment of contour plates for bone gap bridging, along with the utilization of partially meshed plates for bone graft integration, and the incorporation of bioresorbable materials enriched with growth factors, either independently or in combination, warrants consideration. There is a theoretic and empiric benefit of decreased degree of hypoesthesia due to avoiding the dense lateral cortex at the mid body of the mandible, which is the site of most direct nerve injuries in a traditional BSSO [73,74]. This is where the inferior alveolar nerve travels laterally as it approaches the mental foramen. Therefore, redirecting the vertical osteotomy to extend posteriorly will inherently decrease the risk of damage to the nerve. The HSSO also increases the probability of the nerve remaining in the distal segment as there is less surface area of the inferior alveolar canal involved when splitting the mandible. Empirically our results have shown that in over 20 cases using HSSO, nerve sensation appears to return sooner than a traditional BSSO. Further studies are warranted to substantiate these findings. It is critical to undertake comprehensive research to corroborate the diminished involvement of the inferior alveolar nerve and to verify the enhanced stability relative to the traditional inverted L technique, attributed to the greater bony overlap achieved with our technique. Additionally, a thorough comparative analysis delineating the efficacy, benefits, and limitations of HSSO vis à vis angle onlay PSIs is essential.

The HSSO necessitates reduced surgical exposure compared to a conventional BSSO which, in turn, demands less exposure than an inverted L osteotomy. Therefore, we can maintain vascularity and minimize the complications associated with dissecting the pterygomasseteric sling [75,76,77], inferior border periosteum, and muscle attachments. We may, however, elect to cut the pterygomasseteric sling along with the insertion of the sphenomandibular ligament, to help minimize relapse in hyperdivergent cases requiring large counterclockwise rotations [39]. The HSSO has theoretic increased bony overlap in comparison to an inverted L osteotomy, but may be less than a BSSO in some cases. Further studies and long-term follow-up are warranted to validate these considerations and establish the long-term efficacy and stability of this modified approach. It is imperative to note that similar techniques were described in 2017 by Grimaud et al. [78], later in 2019 by Ferri et al. [79] and Mont’Alverne et al. [80], and lastly in 2022 by Castillo and Naranjo [81], which outline a lot of similar advantages. HSSO differs from these techniques for the reasons outlined throughout the paper. It demonstrates optimized versatility for asymmetry cases as we have complete manipulation of the ramus height, gonial angle, and inferior border position. It has shown stability with a dynamic opposing arch form such as a three-piece Le Fort. It has been shown with hemifacial hypertrophy where the ramus height can be shortened without an inferior border reduction. It has shown stability in a case of total idiopathic condylar resorption. It is also not limited by the distance of the inferior alveolar canal to the inferior border, does not require a trocar or posterior plating of the angle, and does not rely on orthodontic extrusion to correct a post-operative posterior open bite as can be carried out in conjunction with a Le Fort. Lastly, our approach aligns with the future workflows of orthognathic surgery, as evidenced by our successful integration of VSP and PSIs.

### Limitations, Drawbacks, and Consideration

The inherent compromise of guided surgery with custom plates, despite their proven stability, precision, and efficiency [82,83,84], is the need for technique-sensitive plating, a challenge that can be mitigated with the use of a right-angle driver. Guided surgery facilitates the use of milled or 3D printed pure titanium plates, featuring longer spans and screws for enhanced rigidity, in contrast to alloy-based non-custom plates susceptible to fractures and functional impairments [58]. Infection rates are similar between patient-specific plates and miniplate fixations [85]. Yet, careful positioning of fixation screws is crucial, as guided methods demonstrate lower root involvement than non-guided techniques [86]. Another disadvantage, particularly in non-guided cases, is leverage created at the angle which can lead itself to an open bite. This is ameliorated by maintaining post-operative orthodontics for a slightly longer period, refraining from using anterior maxilla to posterior mandible elastic vectors for 4–6 months [87]; cutting the sling at the angle to minimize relapse [39], and patient dietary compliance for the first 6 weeks [88].

## 5. Conclusions

We anticipate that our findings will encourage practitioners to consider the Hanna modified sagittal split osteotomy (HSSO) for applicable cases. The HSSO has demonstrated its potential as an alternative to the inverted L osteotomy and BSSO for patient-specific osteotomies. It serves as a solution for facial asymmetry issues such as mandibular asymmetry, hemifacial hypertrophy, hemifacial microsomia, hyperdivergent profiles with anterior open bite, and total idiopathic condylar resorption, among others. Its distinctive capability to be performed entirely transorally, with or without the use of custom plates, while offering improved inferior alveolar nerve safety, concurrently avoiding facial nerve, artery, and vein involvement, and theoretically enhancing stability through increased bony overlap relative to an inverted L, sets it apart from traditional methods.

The HSSO integrates aesthetic considerations with functional enhancements. When compared to the conventional bilateral sagittal split osteotomy (BSSO), our technique exhibits numerous benefits, including reduced dissection of the pterygomasseteric sling, potentially decreased involvement of the inferior alveolar nerve, and enhanced control over the ramus height, gonial angle, and inferior border positions. This eliminates the risk of soft tissue projection of an inferior border step and the necessity for gonial angle implants, preserving or enhancing the aesthetics of the jawline.

The encouraging outcomes of the HSSO, as demonstrated in our case series, highlight its capacity to deliver predictable, functional, and aesthetically pleasing results; thereby offering a viable alternative to techniques such as the inverted L osteotomy and BSSO. Nonetheless, the necessity for further research and prolonged follow-up is paramount to validate these preliminary results and ascertain the long-term efficacy and stability of the HSSO. Ongoing research is vital for the HSSO to gain recognition as a standard practice in orthognathic surgery, thereby advancing personalized patient care within the realm of maxillofacial surgery.

## Data Availability

The data presented in this study are available on request from the corresponding author due to privacy reasons.
